# Individual differences in human frequency-following response predict pitch labeling ability

**DOI:** 10.1038/s41598-021-93312-7

**Published:** 2021-07-12

**Authors:** Katherine S. Reis, Shannon L. M. Heald, John P. Veillette, Stephen C. Van Hedger, Howard C. Nusbaum

**Affiliations:** 1 Department of Psychology, The University of Chicago, Chicago, IL USA; 2 Department of Psychology, Huron University College, London, ON Canada; 3 Brain and Mind Institute, Western University, London, ON Canada

**Keywords:** Auditory system, Human behaviour

## Abstract

The frequency-following response (FFR) provides a measure of phase-locked auditory encoding in humans and has been used to study subcortical processing in the auditory system. While effects of experience on the FFR have been reported, few studies have examined whether individual differences in early sensory encoding have measurable effects on human performance. Absolute pitch (AP), the rare ability to label musical notes without reference notes, provides an excellent model system for testing how early neural encoding supports specialized auditory skills. Results show that the FFR predicts pitch labelling performance better than traditional measures related to AP (age of music onset, tonal language experience, pitch adjustment and just-noticeable-difference scores). Moreover, the stimulus type used to elicit the FFR (tones or speech) impacts predictive performance in a manner that is consistent with prior research. Additionally, the FFR predicts labelling performance for piano tones better than unfamiliar sine tones. Taken together, the FFR reliably distinguishes individuals based on their explicit pitch labeling abilities, which highlights the complex dynamics between sensory processing and cognition.

## Introduction

Research on auditory object perception typically focuses on the cortical networks that organize the recognition process. Whether conceived of as a dual pathway^1^ or focused on pattern classification^2^, the theoretic framing is based on an ascending auditory recognition system in which frequency specific encoding in primary auditory cortex from the eighth nerve is increasingly refined in temporal cortex for abstract sound category classification and recognition. Much of the research on cortical auditory processing suggests that the site of auditory long-term memory and thus the factors that might influence representation and recognition reside in a cortical network^3^. This suggests that while subcortical mechanisms may be important in the ascending auditory pathway, given that these mechanisms operate below cortical memory formation and storage, they are involved in neurally-encoded auditory signal refinement and transmission but not specifically conditioned by experience.

However, research by Kraus and colleagues has suggested a very different view of the functional role of the subcortical ascending auditory system in perception. For example, their research has shown that musical expertise modifies the auditory coding of pitch in a way that benefits learning tone language patterns^4^. In this research, group differences in musical experience are related to the frequency-following response (FFR) for speech stimuli as well as music and thus have generalized beyond the specific context of experience. Moreover, they argue that the group difference in the auditory brainstem response (ABR) due to musical training predicts how the groups learn. While it is unclear if there is descending cortical control over the brainstem response that sharpens it, or whether there is experiential tuning of the FFR from the bottom-up, it is important that by some mechanism, the ascending auditory pathway is not just a passive signal transmission line, but it is changed in processing by experience. Indeed, there is now substantial research showing that experience can alter encoding in the FFR substantially^5–8^, even after a relatively short period of training^9^.

However, it is still not clear whether the observed experience-based changes in the FFR are reflected in behavior. Certainly, if auditory encoding increases the fidelity of the neural representation of frequency, frequency-based auditory performance should improve. Musacchia et al.^10^ observed that neural responses attributed to the brainstem, including the FFR, correlated with scores in certain musical skill tasks (e.g. timbre discrimination). Moreover, Marmel et al*.*^11^ found that aspects of the FFR predict the ability to discriminate between pitches in a forced-choice task. Coffey et al.^12^ found that individual differences in the FFR relate to pitch perception for tones with a missing fundamental frequency. Carcagno and Plack^9^ found FFR changes following training in a pitch discrimination task, but the observed changes in FFR strength were not specific to stimuli that shared relevant characteristics with the trained stimuli, and correlations between FFR strength and performance metrics were nonsignificant. While these studies support the notion that FFR features seem to relate to individual differences in perceptual acuity, the extent to which plasticity in early auditory structures supports cognitive abilities that are critical to behavior, such as categorization, remains an open question.

Absolute pitch (AP) or “perfect pitch” is the relatively rare ability to label a musical note without the aid of a reference note^13^ and can provide a model system for investigating individual differences in the relationship between auditory encoding and human performance. Given that the spectral structure of the FFR suggests that pitch information is successfully transferred from the cochlea to the central nervous system in all listeners^14^, it may be surprising that most humans are unable to easily utilize that information for the categorization of isolated notes. In contrast, relative pitch perception (categorizing notes in relation to other notes) is the norm among musicians. Absolute pitch possessors’ tuning standards can even be shifted after listening to “detuned” music that maintains relative pitch cues^15,16^. The presumed rarity of AP should be striking, as it is comparable to only being able to classify colors by their relationship to other colors and not with consistent labels such as “blue.” Absolute pitch has often been used as a model system for understanding the interplay between genetic and experiential factors in the development of stable cognitive-perceptual skills^17^—this is a largely unexplored parallel to the way in which the scalp-recorded FFR has been used to investigate the role of experience in shaping auditory encoding, something previously thought to be non-plastic. It could be the case that features of spectral encoding in the FFR may vary between listeners who perceive the pitch of notes absolutely rather than in reference to other notes, supporting the different priorities of categorical processes downstream. Given that AP represents a distinct cognitive skill, the ability to categorize notes, it provides an excellent window into the interplay between low-level encoding, reflected by the FFR, and high-level perceptual categorization.

While AP has traditionally been construed as a dichotomous ability, in which subjects either have or do not have AP^17,18^, recent evidence has suggested that AP ability exists along a spectrum, where AP ability is best described as a continuously distributed variable^19^. While there is sizable variance in pitch labelling ability in the general population^20^, variables that predict continuous variation in absolute pitch perception ability are largely unknown and generally viewed as a consequence of cognitive factors rather than auditory ability^21^. The aim of the present study, then, is to investigate the extent to which individual differences in the FFR, reflecting low-level neural auditory encoding of sounds, predicts variation in pitch labelling ability, a higher-level cognitive process.

## Results

### Behavioral results

**Figure 1:**
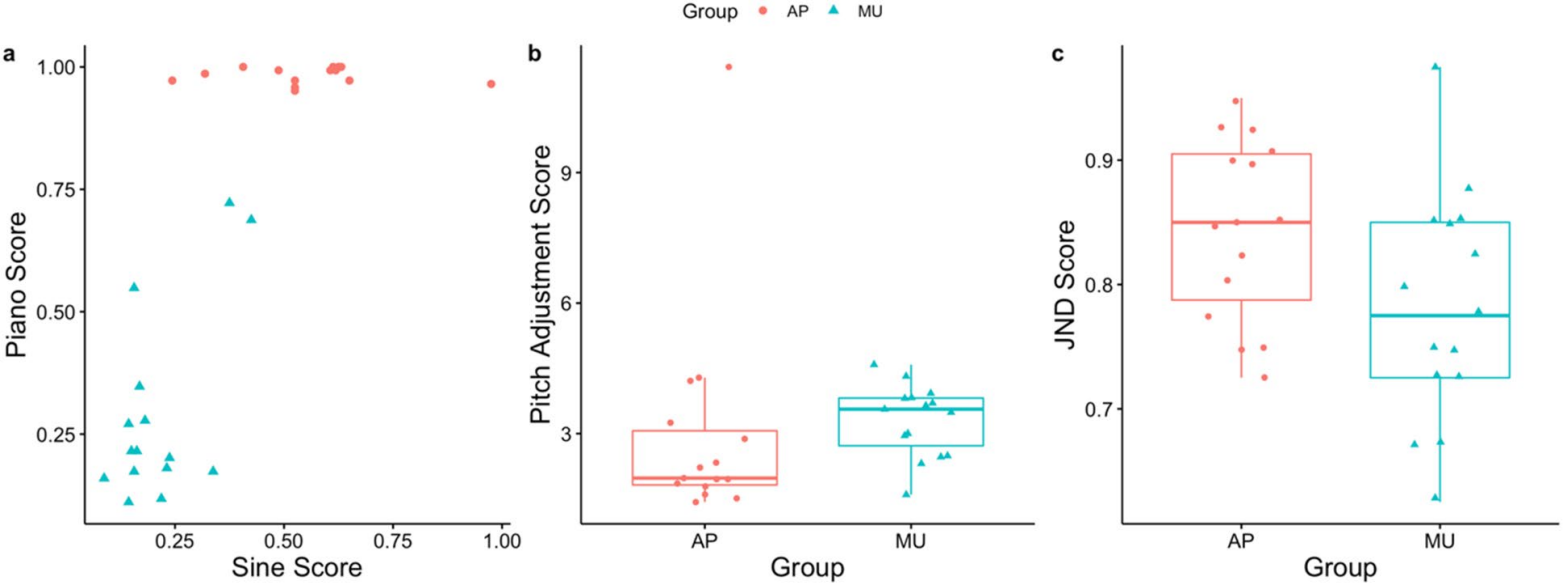
Spread of Behavioral Data. Individual data points are provided for individual subjects. Red circles represent individuals who self-report as an AP possessor, while turquoise triangles represent other musicians. (**a**) Comparison of performance on the AP sine tone conservative measure compared to performance on the AP piano tone conservative measure. (**b**) Performance on the pitch adjustment task. (**c**) Performance on the just-noticeable-difference task.

There was a reasonable spread of performance on pitch performance for sine tones for both self-reported AP possessors (M = 0.554, SD = 0.163) and other musicians (M = 0.212, SD = 0.0960), as well as for piano tones (self-reported AP possessors: M = 0.984, SD = 0.0165; other musicians: M = 0.294, SD = 0.199). See Fig. 1A for a visualization of how the scores relate to one another. The distribution of average pitch labeling ability was approximately M = 0.769, SD = 0.0814 for self-reported AP possessors and M = 0.253, SD = 0.134 for other musicians. Performance on the pitch adjustment task (measures auditory working memory precision by requiring participants to hold in mind a target note for some period of time prior to manually adjusting the final tone to match the target) for self-reported AP possessors was M = 2.978, SD = 2.507, and M = 3.311, SD = 0.822 for other musicians (see Fig. 1B). Finally, just-noticeable difference (JND) task (assesses one’s ability to behaviorally discriminate between two tones of varying frequency) performance for self-reported AP possessors was M = 0.849, SD = 0.0715, and M = 0.782, SD = 0.0918 for other musicians (see Fig. 1C).

While previous research has found that there is a positive relationship between tonal language experience and AP ability^22^, we did not find such a relationship here for both the AP piano tone conservative measure (*t*(11.1) = 0.55, *p* = 0.59) and the AP sine tone conservative measure (*t*(10.5) = 0.74, *p* = 0.48). We also found no significant difference between subjects who identified their primary instrument as fixed-pitch and not fixed-pitch on both performance on the AP piano tone conservative measure (*t*(9.7) = − 0.50, *p* = 0.63), and AP sine tone conservative measure (*t*(9.3) = − 0.66, *p* = 0.53). In other words, effects reported in past research—such as that lessons on piano or other fixed-pitch instruments enhance AP abilities^23^ or that personal musical histories are reflected by individual performance on absolute pitch recognition tasks^24^—are not significantly present in our sample.

### Electrophysiology results and predictive modeling

The FFR to the piano tone (r = 0.26, t(999) = 31.49, *p* = 9.18e-152) and the FFR to the unfamiliar complex tone (r = 0.27, t(999) = 31.91, *p* = 1.31e-154) both predict pitch-labelling performance better than chance, but not significantly differently from one another (t(1994.81) = − 1.19, p = 0.234). Both the piano tone FFR (t(1875.59) = 38.81, *p* = 2.42e-242) and complex tone FFR (t(1840.56) = 39.16) perform significantly better than the speech-evoked FFR (r = − 0.15), which performs significantly worse than chance (t(999) = − -22.71, *p* = 2.29e-92).

The Lasso regression yielded the following sparse models, reported with regression coefficients in normalized units for easy comparison across models. Note, in Eq. (3), that the Lasso regression selected harmonics near the formant frequencies of the spoken /da/ to include in the model; while this is encouraging with respect to the Lasso technique picking out relevant predictors, the speech model does not perform above chance, so we caution against attempting to interpret the presence or absence of particular parameters in the model.

1 $$ {\text{Complex}}\;{\text{tone}}:\quad \hat{y}_{{logit}}  = 6.7 \times 10^{{ - 18}}  - 0.33F_{0}  + 0.017H_{5} $$

2 $$ {\text{Piano}}\;{\text{Tone}}:\quad \hat{y}_{{logit}}  = ~ - 5.1 \times 10^{{ - 18}}  - 0.063F_{0}  - 0.45H_{1}  + 0.28H_{4} $$

3 $$ {\text{Speech}}:\quad \hat{y}_{{logit}}  = ~1.9 \times 10^{{ - 17}}  + 0.15F_{0}  - 0.021H_{6}  + 0.022H_{{12}} $$

The piano tone FFR predicts AP classification performance for both piano tones (r = 0.29, t(999) = 31.11, *p* = 4.11e-149) and sine tones (r = 0.08, t(999) = 12.26, *p* = 2.69e-32). However, the model does predict significantly better on piano tone performance (t(1729.47) = 19.22, *p* = 8.70e-75), suggesting a more specific effect of auditory encoding on pitch classification ability.

4 $$ {\text{Piano}}\;{\text{Tones}}:\quad \hat{y}_{{logit}}  =  - 3.3 \times 10^{{ - 17}}  - 0.013F_{0}  - 0.46H_{1}  - 0.0044H_{3}  + 0.25H_{4} $$

5 $$ {\text{Sine}}\;{\text{Tones}}:\quad \hat{y}_{{logit}}  = 4.4 \times 10^{{ - 17}}  - 0.089H_{1} ~ + ~0.0012H_{4} $$

The frequency-following responses to the piano tone predicts AP performance better than the behavioral measures (age of music onset, tonal language experience, pitch adjustment and just-noticeable-difference scores) are able to (t(1980.05) = − 16.22, *p* = 1.16e-55), with the latter only performing slightly, albeit significantly, above chance (r = 0.09, t(999) = 11.69, *p* = 1.06e-29). Notably, combining the behavioral and electrophysiological predictors (r = 0.21) yields a model that is worse than that based on only electrophysiological predictors (t(1982.98) = − 4.52, *p* = 6.55e-06), but does do better than the behavioral data alone (t(1997.86) = − 12.23, *p* = 3.08e-33). This suggests that the behavioral measures contain little information about pitch labelling ability that is not already captured by the FFR. Interestingly, the behavioral-only model (see Eq. 6) removed all predictors except for the just-noticeable-difference score, a measure of perceptual discrimination ability, indicating that the other behavioral measures do not provide additional information about pitch labelling ability.

6 $$ {\text{Behavioral}}:\quad \hat{y}_{{logit}}  = 8.7 \times 10^{{ - 18}}  + 0.023JND $$

7 $$ {\text{Combined}}:\quad \hat{y}_{{logit}}  =  - 5.2 \times 10^{{ - 17}}  - 0.39H_{1}  + 0.18H_{4}  + 0.20JND - 0.0038age\_onset~ $$

8 $$ {\text{FFR}}:\quad \hat{y}_{{logit}}  =  - 5.1 \times 10^{{ - 18}}  - 0.063F_{0}  - 0.45H_{1}  + 0.28H_{4} $$

## Discussion

Though previous work has shown that individual changes in the FFR can arise as a result of past experience, such as musical training, the exact relationship between the FFR and behavior has remained ambiguous. Individual differences in the FFR have been related to performance on certain perceptual discrimination tasks^12^ and such differences have been shown to emerge following training in such a task^9^, but these individual differences were not specific to task-relevant spectral features and studies that relate auditory encoding to performance rarely compare the magnitude of FFR differences across stimuli from different domains. This omission is particularly problematic, as many known FFR effects persist across auditory domains; for example, musical training seems to impact the FFR encoding of speech sounds, leading some researchers to argue that experience-dependent changes in the FFR are generally domain-nonspecific^25^.

The present study provides compelling evidence for the domain specificity of individual differences in FFR spectral features. While our data replicate previous findings that FFRs to domain nonspecific stimuli can predict scores in an auditory task, as the predictive performance of our model deviates from chance for all stimuli, we find robust differences between the predictive power of FFRs to different stimuli. We find that the FFRs to tones predicts performance substantially better than to speech stimuli, seemingly corresponding to the subjects’ experience attending to the pitch of notes regardless of the familiarity of their timbres. In contrast, the FFR to the piano tone, a familiar timbre, does not seem to predict pitch-labelling ability for piano tone stimuli any better than the FFR to the complex tone, so instrument-specific advantages in brainstem encoding do not seem to account for well documented own-instrument advantage effects in the AP literature^23,24^. Our subjects do, however, generally perform better on the piano tones than on the sine tones, consistent with past literature, so the observed timbre-familiarity advantage may originate from later auditory processing or during subsequent categorization.

Importantly, we find that the FFR to the piano tone predicts subjects’ ability to label the pitch of piano tones significantly better than it does the pitch of sine tones. This finding points toward a view of FFR plasticity as a mechanism that can support domain-specific auditory skills above and beyond the domain-general effects previous researchers have observed.

Notably, individual differences in early sensory encoding, as reflected by the FFR, are able to predict continuous variance in AP ability. Since the variation in pitch labelling ability has largely gone unexplained since researchers have argued that AP should be considered as a graded (rather than dichotomous) ability^20^ this finding is novel. It has long remained an open scientific question why humans can place some types of stimulus characteristics into stable, barely changing categories (such as color) but less so others (such as pitch); understanding the relationship between individual differences in low-level sensory coding and in the higher-level cognitive ability to consistently categorize perceptual stimuli promises to shed light on broader theories of semantic memory, concepts, and categories^26^.

It is tempting to conclude that the mechanism for our observed effect is a difference in stimulus encoding in subcortical structures that covaries with AP ability; indeed, this is how the FFR literature has historically interpreted such results^7,10,25^. Of course, our ability to draw definitive conclusions from our results is limited by the nature of a between-subject design in noninvasive electrophysiology studies using correlation. A predictive relationship between the scalp-recorded FFR and AP ability need not be caused by a true change in auditory encoding in the FFR’s source structures; since part of the FFR is thought to originate subcortically, any anatomical difference between those far-field sources and the recording electrode that covaries with AP ability^27^ could mediate the observed effect by altering volume conduction through the brain. However, such an anatomical difference would affect the scalp recorded FFR similarly for different stimuli, and we observe robust differences in predictive power between stimuli. Individual differences in brain anatomy could conceivably have a compounding influence on some true effect if, for example, changes in white matter density or microstructure, which may affect volume conduction, support higher fidelity phase locking to the acoustic stimulus. While this situation would suggest some true effect exists, it makes estimating the effect size from a scalp-recording tenuous, since the true effect could be correlated with a confounding factor. Lastly, since the FFR is now thought to originate from a distributed network of cortical and subcortical sources rather than solely from the auditory brainstem as previously thought^28^, a differential contribution of cortical sources, close to the recording electrode, and subcortical sources could account for any attenuation or amplification of power in the FFR. It seems difficult to tease apart this alternative from the traditional explanation with the minimalist recording montage used in most FFR experiments, but this distinction may be addressable in future research using high density electrode montages^29^. Nonetheless, a shift in the relative contribution of different source regions, rather than an overall change in phase-locking to the stimulus, would still speak to the overall hypothesis that differences in early auditory encoding support higher-level cognitive abilities in a domain-specific manner.

The fields of FFR research and AP research share a common interest in how long- and short-term experience interact with less malleable aspects of nervous system development, such as genetics, to alter the encoding of sound. While the mechanisms of AP have traditionally been construed as cognitive, the present study suggests that real variance in pitch labelling ability may be attributable to low-level sensory encoding differences, as reflected in the FFR^30^. Conversely, individual differences in the FFR appear to be much more dependent upon the development of specialized skills and the particular domain of auditory experience than previously thought. As many fields in the behavioral sciences are now discovering, it may not be possible to fully understand cognition or perception without considering their dynamic interaction.

## Materials and methods

### Participants

Thirty-five individuals participated in the experiment, four subjects were removed (one for non-compliance on tasks, one for hardware issues at the time of experimentation, one for failure to meet hearing criteria, and one for a pre-existing neurological condition). Absolute pitch possessors (N = 16) and musically matched subjects (N = 15) were recruited from the Chicagoland area. By including subjects that are expected to show a range of pitch perception ability, we hope that our sample is representative of the population distribution of absolute pitch ability described by Van Hedger et al.^20^. Of the 31 remaining subjects, which included both males and females (16 females) with varying amounts of musical training, the average age was M = 21.6, SD = 3.01. The self-reported absolute pitch possessors reported to play an instrument for M = 15.88, SD = 3.77, years, while the other musicians reported to play an instrument for M = 14.73, SD = 4.48, years (t(27) = 0.765, *p* = 0.451). Three self-reported absolute pitch possessors and seven musically matched subjects were tonal language speakers. 13 self-reported absolute pitch possessors and 10 musically matched subjects identified their primary (synonymous here with first) instrument as being a fixed-pitch instrument (piano).

The study procedure was approved by the Social and Behavioral Sciences Institutional Review Board at the University of Chicago, and all research was performed in accordance with such guidelines. Informed consent was received from each subject.

### FFR acquisition and preprocessing protocol

All recordings were conducted in a soundproof semi-electrically shielded booth. Brainstem electroencephalography recordings were collected while participants were presented with auditory stimuli that were presented binaurally via fitted earbuds attached to Etymotic Research ER-3a insert tube phones at 65–75 dB. Alternating polarity presentation was used to reduce the presence of the cochlear microphonic (CM) in the recorded signal. Each stimulus type was presented 3000 times, 1500 times for each polarity. During recording participants were allowed to watch a silent film, as is common for ABR studies^31^. Stimuli were presented using Psychtoolbox (Matlab Psychtoolbox-3; psychtoolbox.org).

Horizontal montaging^32^ was used using Ag–AgCl electrodes. Electrode placement included a ground electrode on the center of the forehead, an active electrode placed at Cz, and linked reference electrodes placed on both the left and right mastoid. Impedances from Cz, each mastoid individually, and the mastoids together were taken prior to experimentation, with a maximum of 5 k Ohms allowed. BrainVision PyCorder software (BrainProducts) was used to record brainstem responses with an online filter of 0.1 to 3000 Hz.

Preprocessing in BrainVision Analyzer 2.2.0 proceeded as follows. Filtering parameters were dictated by the properties of the stimuli. The EEG recordings in response to the piano and complex stimuli were bandpass filtered (Butterworth 12 dB octave roll-off) from 100 to 2000 Hz, whereas /da/ stimuli were bandpass filtered from 70 to 2000 Hz. All stimuli had an additional notch filter of 60 Hz applied.

We then applied an absolute threshold detection (± 700 mV) on the recorded audio channel via a Boolean expression that selectively finds the negative and positive peak of the start of a stimulus, and marks whichever occurs first. It is vital to use an absolute threshold rather than solely a positive or negative threshold in order to not correct for phase differences between inverted and non-inverted stimuli. By preserving such phase differences, we are able to shift our analysis to mainly examine the ABR portion of the recorded signal rather than the cochlear microphonic (CM), as the ABR is insensitive to phase differences while the CM is not. Segmentation procedures were dependent on the length of the stimulus. Piano and complex tones were 200 ms in length, and the /da/ stimulus was 80 ms in length. As a result, piano and complex segments were defined to start 50 ms prior to stimulus onset and last 250 ms post stimulus onset, /da/ segments were defined to start -10 ms prior to the stimulus onset and last 120 ms post stimulus onset.

Trials that had been contaminated by unwanted artifacts (those that exceeded a strict amplitude threshold of 35 µV) were removed from the dataset. A baseline correction transformation was performed on the 10 ms preceding the /da/ stimulus, and 50 ms preceding the piano/complex stimuli.

### Stimuli

The piano stimulus was sampled from an acoustic piano and produced with Reason software (Propellerhead, Stockholm). The complex tone was generated in Adobe Audition, and the /da/ stimulus was generated by the implementation of a Klatt synthesizer. The fundamental of the complex tone was 207.65 Hz (G#_3_). The fundamental of the piano tone was 261.63 Hz (C_3_). The F0 of the /da/ was 100 Hz. The complex tone stimulus had a fundamental frequency of 207.65 Hz, and consisted of the 3rd, 7th, 8th, and 10th harmonics. An F0 of 100 Hz for our speech stimulus was based on prior auditory brainstem work^10^, and we chose fundamental frequencies for our piano and complex tone stimuli that were in a comfortable middle octave for music listening and is conveniently within the register of most commonly played instruments.

### Prescreening

Participants were administered a sixty second hearing screening using a Welch-Allyn Otoscope equipped with an audiometer. Participants had to detect the occurrence of four tones (500, 1000, 2000, and 4000 Hz), which were presented at random intervals to prevent guessing. Participants were also checked via otoscope to make sure their ear canals were free from debris and that their eardrums were intact.

### Experimental design and statistical analyses

**Figure 2:**
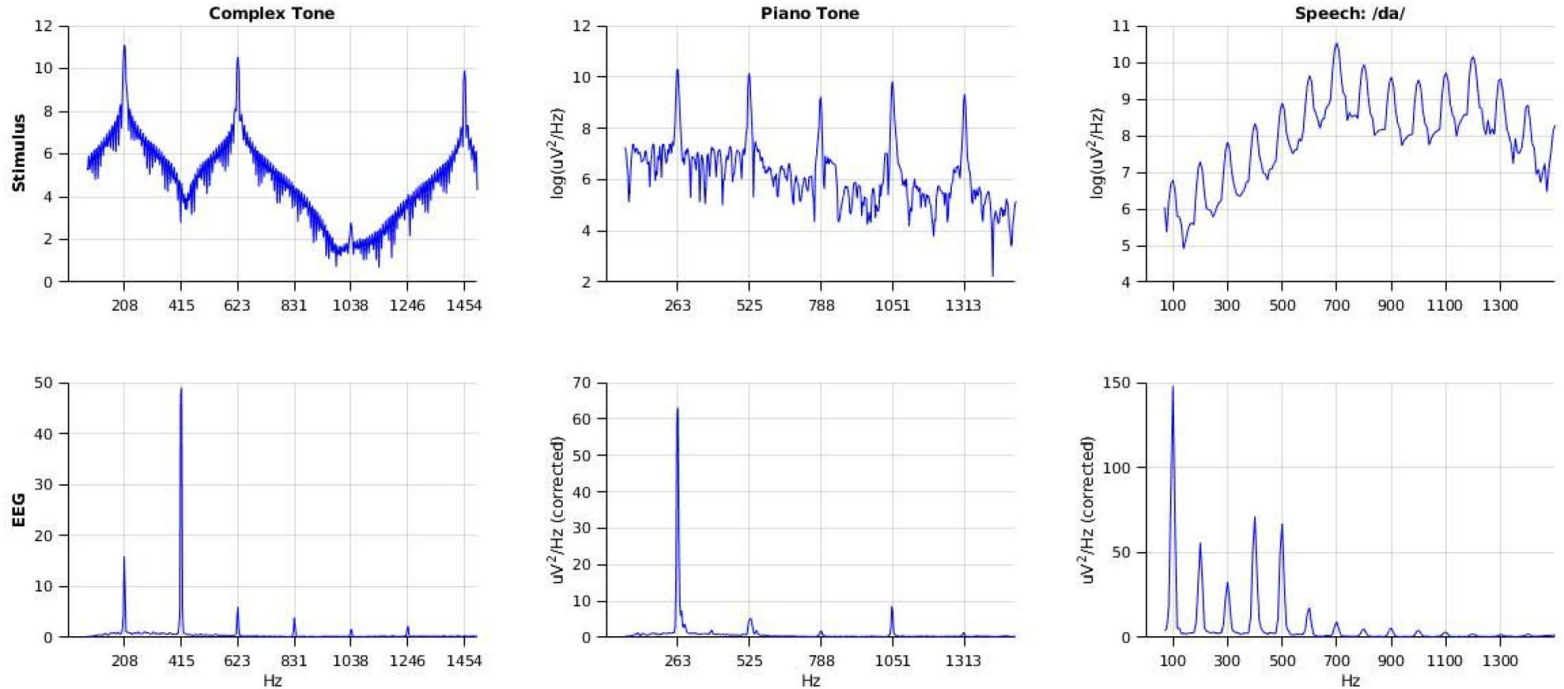
Power Spectra of Stimuli and of Frequency-Following Responses. The nearest integer frequency to the harmonics of the stimulus is marked on each plot, except for the speech stimulus, in which every other harmonic is marked to avoid visual clutter. The EEG spectra are corrected for 1/f frequency drop-off here for visualization, but uncorrected values were used for analysis.

For each subject, we began the experimental session with several questionnaires, where we assessed their musical experience (Absolute Pitch Questionnaire and Musical Experience Questionnaire) and tonal language experience (Language Experience Questionnaire). Afterwards, participants were screened for normal hearing. (Air conduction thresholds < 40 dB, see Prescreening subsection) We then recorded EEG responses to a piano tone, a complex tone with an unfamiliar timbre, and a spoken /da/. (See Stimuli and FFR Acquisition Protocol subsections, above, for more details and Fig. 2 for stimuli power spectra.).

**Figure 3:**
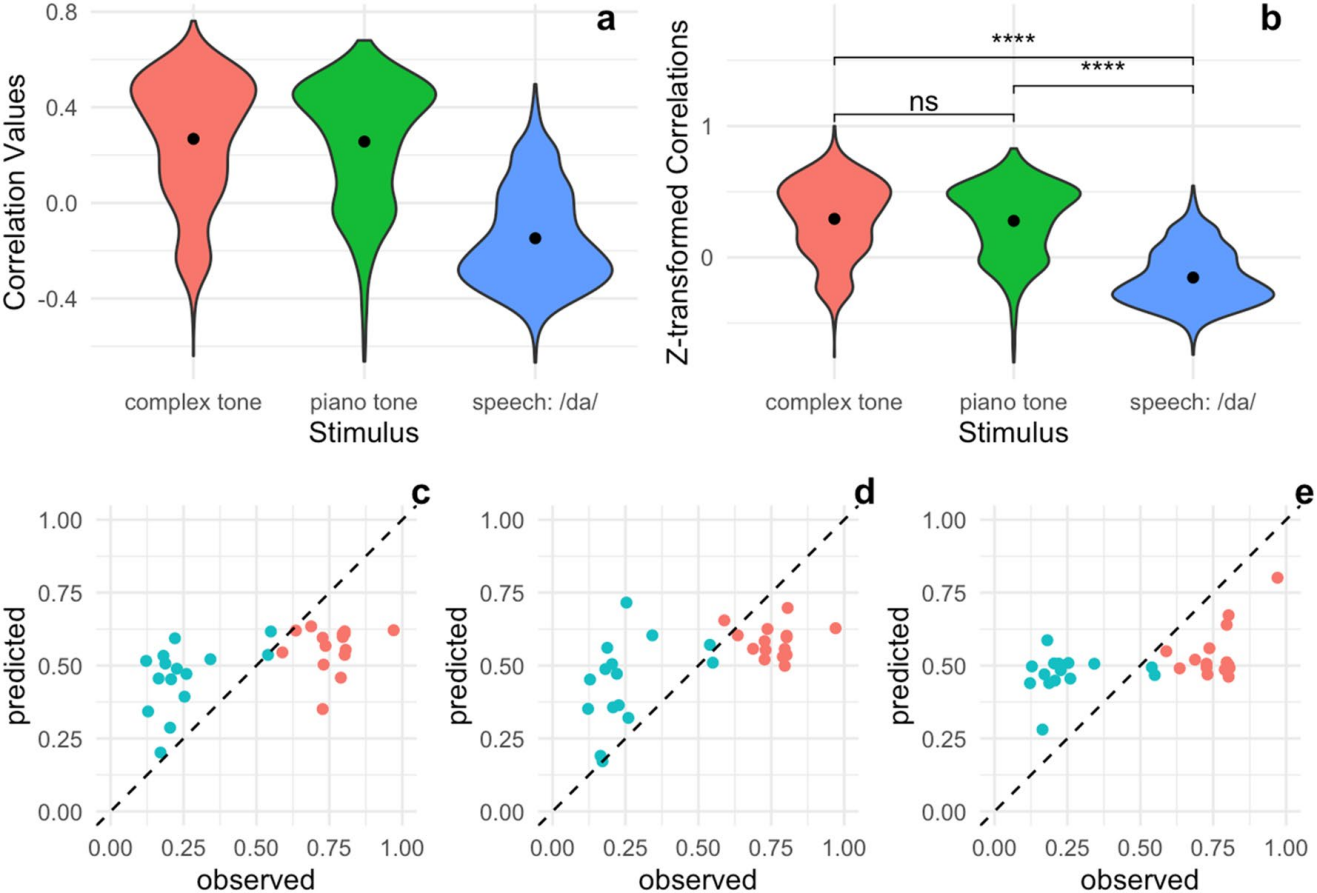
Performance of Lasso Regression Models Using FFR to Different Stimuli as Predictors. (**a**, **b**) For each model, a correlation between the model’s predictions and true AP sine and piano performance was computed on a test set (data points not seen by the model during training) for each of 1000 cross-validation runs as an estimate of how well the model generalizes. See Eqs. (1–3) for final model specifications. (**c**) Predicted AP sine and piano performance values based on complex tone FFR plotted against actual, observed AP performance. Red dots represent subjects who self-reported as AP possessors. (**d**) Predicted AP performance values based on piano tone FFR plotted against observed AP performance. Red dots represent subjects who self-reported as AP possessors. (**e**) Predicted AP performance values based on speech /da/ FFR plotted against observed AP performance. Red dots represent subjects who self-reported as AP possessors.

**Figure 4:**
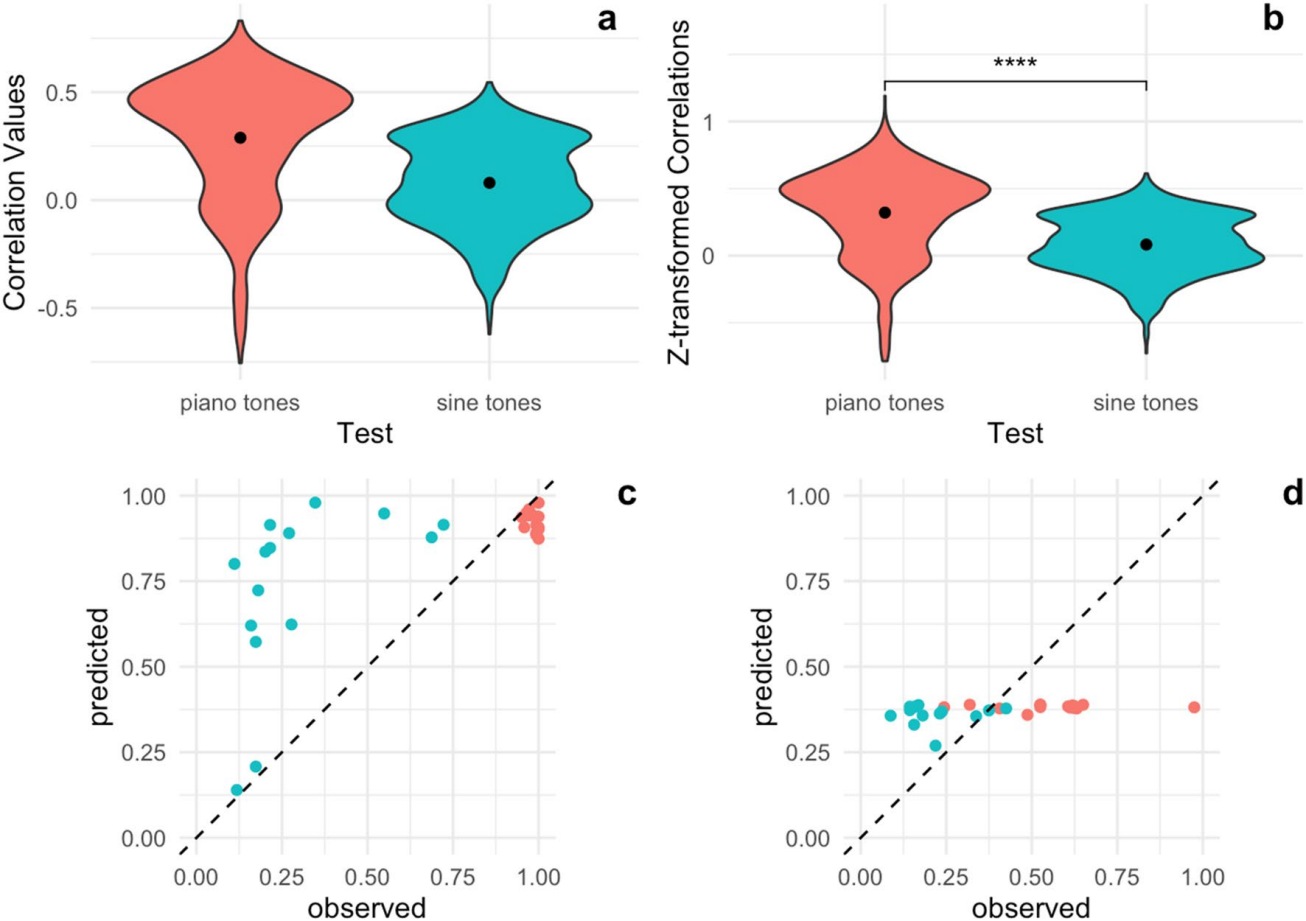
Predictive Performance of Piano FFR on Sine Tones and Piano Tones separately. (**a**, **b**) Correlation between the predicted pitch labelling performances and the true pitch labelling performances on a test set are shown for 1000 cross-validation runs. The FFR to the piano tone predicts pitch labelling performance for piano tones better than it does for sine tones. (**c**) Predicted pitch labelling performance on the piano tones plotted against actual, observed pitch labelling performance. Red dots represent subjects who self-reported as AP possessors. (**d**) Predicted pitch labelling performance on the sine tones plotted against observed pitch labelling performance. Red dots represent subjects who self-reported as AP possessors.

Then, each subject completed an explicit pitch labelling (AP) assessment. The AP assessment consisted of two different paper-pen AP tests. Both tests presented tones across a range of different octaves. The average score of these two tests is what we refer to here as the AP test score, or pitch labelling ability (see Fig. 3C–E for full distribution of AP test scores, and Fig. 4C,D for the performance distribution broken down by piano and sine AP scores). Presentation of the stimuli was controlled by E-prime software.

Subjects subsequently completed a just-noticeable-difference (JND) assessment, which was used to examine how well participants could behaviorally discriminate between two tones. Tones were presented in four blocks of 20 trials each. A standard 1000 Hz tone was used, and in the first block, one of the notes deviated by 56 cents from the 1000 Hz tone. In the second block, the notes deviated by 28 cents, in the third block the notes deviated by 14 cents, and in the fourth block the notes deviated by seven cents. On half of the trials the two tones presented were the same 1000 Hz tone. For a given trial, participants needed to determine whether the two tones were the same 1000 Hz tone or if they were two different tones. This assessment was also graded on a 100% scale. Individual differences in JND task performance should reflect differences in fine grained pitch processing. This task was administered using E-prime software.

Subjects then performed a pitch adjustment assessment (administered using MATLAB), which was based on a task reported by Heald et al.^33^. In this task, participants were required to adjust the frequency of a probe sine tone to match a previously presented target sine tone. The target tone was briefly presented (200 ms) and then immediately masked by noise (1000 ms). Following the noise, a secondary tone (200 ms) was presented. The participants were then asked to try to adjust the secondary tone to match the target tone by adjusting the pitch either up or down. Ten target tones were tested from 471.58 Hz (end point − 80 cents B4) to 547.99 Hz (end point + 80 cents C5), across the B4 and C5 categories. Participants either started above or below these categories (i.e., the location of the secondary tone). Participants were able to adjust the probe tone by adjusting the pitch drawn from a stimulus series. They could adjust the probe either by 10 or 20 cent steps. Given the masking of the target tone, matching performance on this task is designed to measure auditory working memory precision, as it is necessary for participants to hold in mind the target note despite the white noise and intermediary adjustment tones. This interpretation of this task is similarly held by Kumar et al.^34^ and Van Hedger et al.^21^.

The FFR was computed from the EEG responses as follows. Preprocessing was done using BrainVision Analyzer 2.2.0. (See *FFR Acquisition and Preprocessing Protocol* subsection above.). This preprocessed data was then exported from BrainVision Analyzer 2.2.0 to .mat files. (All analyses after this point were scripted in MATLAB and in R; all code, from preprocessing to the generation of figures, can be found at https://github.com/apex-lab/ap-ffr.) In order to maintain an equal number of trials for inverted and noninverted stimuli, we randomly subsampled trials from whichever stimulus polarity (inverted or noninverted) had more trials so that, for each subject, we were left with an equal number of trials of each polarity. Then, all remaining trials (of both polarities) were averaged for each subject and stimulus type (piano, complex tone, speech) to obtain the FFR. This is frequently recommended in the FFR literature^35^ for the purpose of averaging out any stimulus artifact and attenuating the contribution of the cochlear microphonic (see *FFR Acquisition and Preprocessing Protocol* subsection). Next, we applied a Hanning taper to the window corresponding to the duration of each stimulus and computed the power spectrum of each FFR over that window. We then exported the power of each subject’s FFR at each harmonic of its eliciting stimulus (up to 1500 Hz, see Fig. 2) for analysis in R. (These files are available for researchers who wish to reproduce our analyses.)

We then assessed whether the FFRs elicited by stimuli from a variety of auditory domains (piano, speech, and a novel complex periodic signal) were predictive of pitch labelling performance on the score (accuracy) of both AP tests. The reason for focusing on predictive performance, rather than relying on null hypothesis significance testing for inference, is that in principle all the harmonics of a stimulus (and thus the FFR) contain information about pitch. In order to avoid making any assumptions about which harmonics to include but not allow our analysis to suffer from problems inherent to high-dimensional regression (the “curse of dimensionality,” Friedman, 1997)^36^, we employed the Lasso regression technique to fit sparse generalizable models to our data. We describe the Lasso regression technique in some detail below in the *Model Fitting* subsection below.

First, we fit separate models for each FFR eliciting stimulus, predicting the pitch labelling ability across both AP tests (sine and piano tones). Pitch labelling ability is operationalized by awarding 1 point for correctly labelling a note and 0.75 points if only a semitone off, then dividing total points awarded by the number of trials. This is considered a relatively conservative measure, specifically with regard to identifying intermediate AP possessors, and has been used by a number of influential studies^18,37,38^. However, alternative measures of AP ability, such as mean absolute deviation (MAD) in semitones and raw accuracy, are provided for interested researchers in our open dataset. (Though we found the reported results were robust to the operationalization of AP.) Since this measure is [0, 1] bounded, we logit transform it before fitting the model. For each model, we compute the correlation between model predictions and true pitch labelling ability on a test set for each of 1000 cross-validation runs. We then apply the Fisher *z-*transformation to these *r* values (since they would otherwise be [0, 1] bounded and therefore non-normal) and compare each model’s performance to chance (*r* = 0) with a one-sample *t-*test. We also compare the three models to one another to test whether the auditory domain of the FFR eliciting stimulus matters when predicting pitch labelling performance. Full distributions of raw and transformed *r* values are reported (Fig. 3), and regression coefficients (fit on the full dataset) are reported in normalized units for easy comparison between models.

In order to assess the evidence of a specific effect of low-level auditory encoding on task performance, we then separately fit models predicting pitch labelling performance on sine tones and pitch labelling performance on piano tones from the piano elicited FFRs. We compared these models to chance and to each other using *t* tests on the *z-*transformed *r* values from 1000 cross-validation runs. The full distribution of *r* values is reported in Fig. 4.

In total, we report 12 statistical tests. In order to control for multiple comparisons, we apply a Bonferroni correction, resulting in a new significance threshold of α = 0.00417 against which the reported *p*-values should be compared.

### Model fitting

While ordinary least squares regression finds regression coefficients β to minimize the loss function $SSE\left( \beta  \right) = \mathop \sum \limits_{i} \left( {\hat{y}_{i}  - y_{i} } \right)^{2}$, where $\hat{y}$ is what the model predicts, Lasso regression minimizes $L\left( \beta  \right) = SSE\left( \beta  \right) + ~\lambda \mathop \sum \limits_{j} \left| {\beta _{j} } \right|$. The addition of a penalty term for the size of β means that the fit model will only include nonzero values of β (regression coefficients) if the increase in the penalty term is offset by enough of a decrease in the sum of squares error (SSE). In order to ensure that results are *generalizable,* we pick λ (which determines how much the model will “care” about the penalty term) to maximize model performance on data that the model never saw during training (a hold-out set). This ensures that the model only includes predictor variables that robustly help it predict *new* data (the predictors that we can expect to generalize outside of our particular sample to the target population), setting the coefficients for all other predictors to zero. In exchange for performing near-optimal variable selection for us, Lasso regression does not provide a *p*-value for each remaining regression coefficient, but we can derive a *p*-value for the full model by comparing model performance on a test set (more data points the model did not see during training) to chance. This *p*-value, arguably, is more meaningful than those traditionally reported since it is derived from a measure of how well a model *generalizes* to new data, while *p-*values for ordinary linear regression are more prone to reach significance just because of noise within the sample. For more detail on the theory and practical implementation of the Lasso, see James et al.^39^.

Each time we fit a model we are actually fitting many models. First, we divide the data randomly into a training set (2/3 of the data) and a test set (the remaining 1/3 of the data). Next, we train models using many different values of λ (from 0.01 to ${10}^{10}$) and select the model that minimizes the leave-one-out cross-validation score over the training set. We then compute the performance of this model on the test set (picking the metric of our choosing as a “cross-validation score,” in our case $r = {\text{corr}}\left( {\hat{y},~y} \right)$) as a measure of how well the model predicts new data.

**Figure 5:**
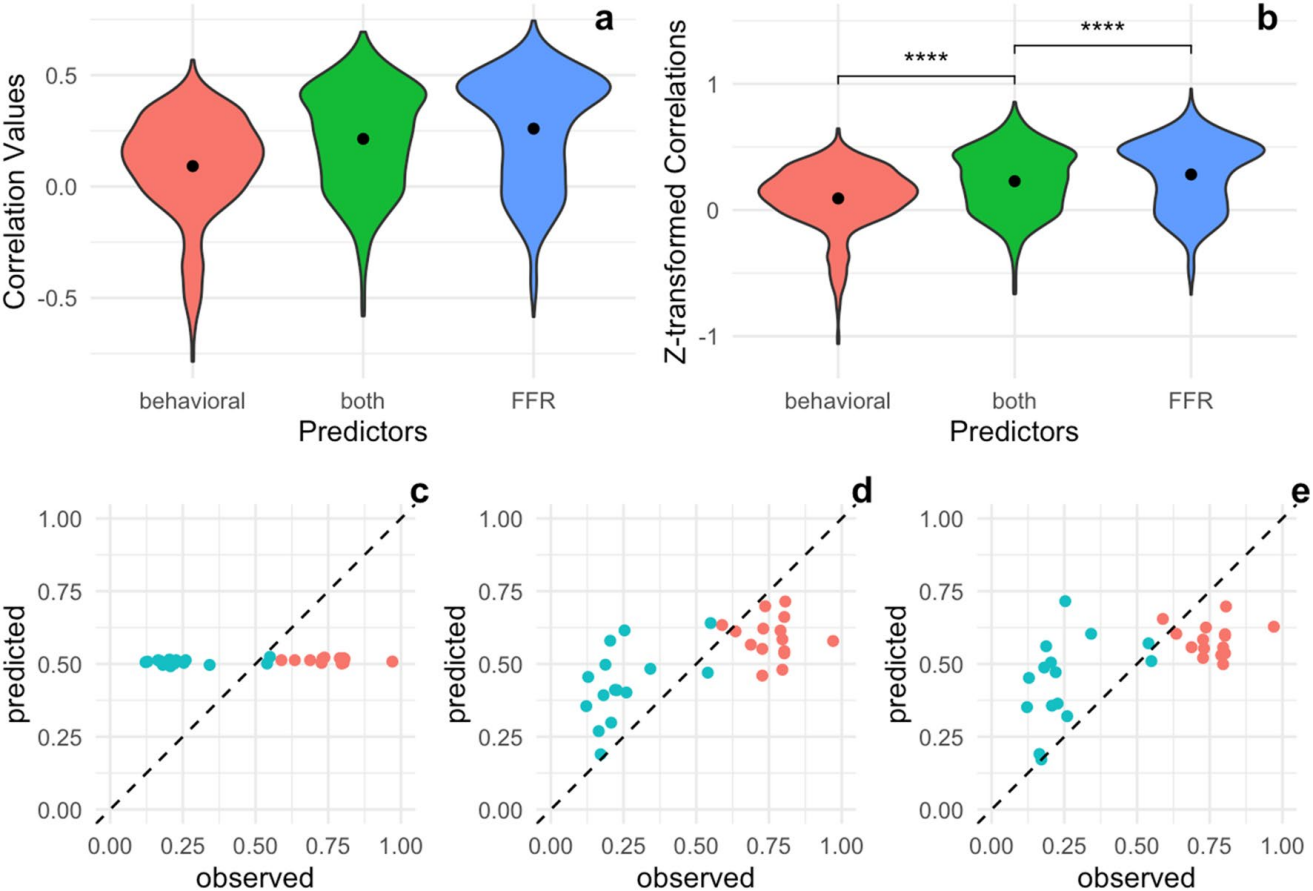
Predictive Performance of Behavioral Tests, Piano FFR, and a Combined Model on Pitch Labelling Performance. (**a**, **b**) Correlation between the predicted pitch labelling performances and the true pitch labelling performances on a test set are shown for 1000 cross-validation runs. The FFR to the piano tone predicts pitch labelling performance better than the behavioral tests as well as the combined model. (**c**) Predicted pitch labelling performance based on the behavioral tests plotted against actual, observed pitch labelling performance. Red dots represent subjects who self-reported as AP possessors. (**d**) Predicted pitch labelling performance based on a combined model of both behavioral tests and the piano FFR plotted against observed pitch labelling performance. Red dots represent subjects who self-reported as AP possessors. (**e**) Predicted pitch labelling performance based on the piano FFR plotted against observed pitch labelling performance. Red dots represent subjects who self-reported as AP possessors.

If using the cross-validation score for inference, one has to be concerned about whether performance on the test set may have been good (or bad) by mere chance, and as it happens, the random choice of test set can result in dramatically variable cross-validation scores (see Figs. 3, 4, 5). To account for this variability, we repeat this whole cross-validation procedure 1000 times for each model, each with a new, random training-test split, and report the full distribution of *r* values generated.

## Data Availability

The analysis code is available at https://github.com/apex-lab/ap-ffr, and the data used in our analyses is available on Open Science Framework with https://doi.org/10.17605/OSF.IO/HRCVS.
